# Molecular Epidemiological Survey of Porcine Rotavirus in the Guangxi Region from 2020 to 2025 and Isolation and Identification of the G9P[23] Strain CH-GXGL-PoRV-3151-2021

**DOI:** 10.3390/vetsci13070631

**Published:** 2026-06-29

**Authors:** Shuo Zhao, Xianhua Wu, Ying He, Jinmu Lin, Xinlin Zhong, Baojiang Lin, Wen Zhao, Xinting Xu, Qunpeng Duan, Xunye Yang, Han Shao, Ying Peng, Yilan Xu, Tingting Chen, Chenyu Quan, Bingxia Lu, Wenfeng Wang, Yang Qin, Zhongwei Chen, Yangqing Lu, Yibin Qin

**Affiliations:** 1 Animal Science and Technology College, Guangxi University, Nanning 530004, China; zhaoshuo5500@163.com (S.Z.); wuxianhua5660@163.com (X.W.); 2498262507@qq.com (X.Z.); 769246915@qq.com (W.Z.); 492873304@qq.com (Q.D.); 407700579@qq.com (H.S.); 2 Key Laboratory of Veterinary Biotechnology of Guangxi, Guangxi Veterinary Research Institute, Key Laboratory of China (Guangxi)-ASEAN Cross-Border Animal Disease Prevention and Control, Ministry of Agriculture and Rural Affairs, Nanning 530001, China; heying921@163.com (Y.H.); xuxtisabel@163.com (X.X.); yangxunye@163.com (X.Y.); 3274167685@qq.com (Y.P.); 1261184469@qq.com (Y.X.); 1246349356@qq.com (T.C.); 290917041@qq.com (C.Q.); lubingxia13@163.com (B.L.); 1406240968@qq.com (W.W.); 1756587992@qq.com (Y.Q.); chen_zhong-wei@163.com (Z.C.); 3 Guangxi State Farms Yongxin Animal Husbandry Group Shengtang Animal Husbandry Co., Ltd., Liuzhou 545211, China; 4 People’s Government of Xinjing Town, Baise 533899, China; 15578170986@163.com; 5 Guangxi Reclamation Yongxin Animal Husbandry Group Xijiang Co., Ltd., Guigang 537104, China; 18078562721@163.com

**Keywords:** porcine rotavirus, molecular epidemiology, genotyping, virus isolation, phylogenetic analyses

## Abstract

Porcine rotavirus (PoRV) has emerged as a primary pathogen causing viral diarrhea in pigs, resulting in significant economic losses. This study was conducted to systematically characterize the epidemiology and genotypic characteristics of PoRV in Guangxi, China. A total of 870 diarrheic pig samples were collected from Guangxi during 2020–2025. The qRT-PCR results indicated an overall PoRV-positive rate of 41.38% (360/870), and the annual positivity rate showed an overall upward trend. The genetic evolutionary analysis of the VP4, VP6, and VP7 genes indicated that PoRV predominantly belonged to the A group and the predominant P genotype observed was P[13] (76.83%), while the G genotypes were G5 (36.56%) and G9 (33.33%). The most prevalent genotype combinations were G9P[13]I5 and G5P[13]I5. CH-GXGL-PoRV-3151-2021, a PoRV strain isolated from positive samples, was identified via RT-PCR, qRT-PCR, whole-genome sequencing, and IFA. This strain was assigned the 11-segment genotype constellation G9-P[23]-I5-R1-C1-M1-A8-N1-T1-E1-H1 based on whole-genome sequencing. NSP1 and NSP2 showed high similarity to human rotavirus strains, whereas VP1–VP4, VP6, VP7, and NSP3–NSP5 showed high similarity to porcine rotavirus strains. This study indicates the widespread circulation of PoRV in Guangxi, with multiple G genotypes, including G9, G5, G4, G3, G2, and G26, being detected. The isolated G9P[23]I5 strain exhibits the same genotype as the strains that have become increasingly prevalent in recent years. This strain may represent a possible reassortant between porcine and human rotaviruses. This study offers significant insights into the epidemiology of PoRV and the prevalent genotypes in Guangxi, thereby supporting the development of targeted prevention strategies and novel vaccines.

## Simple Summary

Rotavirus (RV) is a zoonotic pathogen that causes acute diarrhea in animals and infants. Within the context of the pig farming industry, it frequently co-infects with other diarrhea-causing pathogens, resulting in significant economic losses. From 2020 to 2025, nucleic acid testing for porcine rotavirus (PoRV) was conducted on 870 swine diarrhea samples from Guangxi, resulting in a positivity rate of 41.26%. The predominant genotypes were P[13] for VP4, I5 for VP6, and G5/G9 for VP7, with G4 being also frequently detected. A multitude of genotype combinations and diverse sources were identified concurrently. The strain CH-GXGL-PoRV-3151-2021 was successfully isolated from these samples and may represent a possible porcine–human reassortant rotavirus. The findings of this study provide reliable data for understanding the epidemiology of PoRV and its novel vaccine development, underscoring the importance of sustained surveillance for this significant porcine disease agent.

## 1. Introduction

Porcine rotavirus (PoRV) is a highly contagious enteric virus that causes vomiting and watery diarrhea in infected pigs. Its high transmissibility and propensity to co-infect with other swine diarrhea pathogens have rendered it a significant concern for the swine industry [1]. PoRV can infect pigs of all ages. The incidence and mortality rates of the virus can reach 50–100% in newborn piglets aged 1–5 days. Infection in pigs of other growth stages has been demonstrated to result in growth impairment or subclinical infection [2,3]. It has been observed that certain PoRV strains contain gene fragments that exhibit a high degree of similarity to those of human rotavirus, suggesting the potential for human infection [4]. Consequently, research into PoRV is of crucial importance not only for the control of piglet diarrhea but also for the advancement of human public health.

PoRV is a non-enveloped, double-stranded RNA virus with a genome size of approximately 18.5 kb, composed of 11 discrete linear RNA segments [5]. The genome encodes six structural proteins (VP1, VP2, VP3, VP4, VP6, and VP7) and five non-structural proteins (NSP1, NSP2, NSP3, NSP4, and NSP5). Based on the inner capsid protein VP6, PoRV can be divided into 10 distinct groups (A–J). Group A rotavirus is one of the major rotavirus groups associated with diarrhea in both piglets and infants [6,7,8,9]. The genes encoding the outer capsid protein VP7 and the spike protein VP4 allow PoRV to be further subdivided into 27 G types and 38 P types, enabling preliminary dual-system classification [10]. Advanced studies have developed a new classification system based on whole-genome homology and segment-specific homology thresholds, enabling analysis of PoRV’s host range, replication, and virulence genes. This approach involves subdividing strains into distinct genotypes through genetic classification and evolutionary analysis [11,12,13]. The new genotyping system is represented as Gx-Px-Ix-Rx-Cx-Mx-Ax-Nx-Tx-Ex-Hx, denoting VP7-VP4-VP6-VP1-VP2-VP3-NSP1-NSP2-NSP3-NSP4-NSP5/6, respectively [14]. However, it is noteworthy that the classification of these fragments as belonging to the same genotype is contingent upon the attainment of specific thresholds for the nucleotide identity, with the threshold values being 80%, 80%, 85%, 83%, 84%, 81%, 79%, 85%, 85%, 85%, and 91%, respectively [15].

An analysis of epidemiological data from both domestic and international sources reveals a worrisome trend: the prevalence of PoRV is escalating. In a previous study, a global epidemiological analysis of PoRV was conducted across five continents, with China excluded, from 2005 to 2022. The analysis revealed that the prevalence rate rose from 23.99% to 47.02% [16]. In recent years, diarrhea outbreaks in piglets caused by PoRV have exhibited a high incidence rate, and this virus has become the predominant cause [17,18]. In the nascent stages of PoRV prevalence, the G5 and P7 genotypes exhibited a high degree of predominance [18,19,20]. However, the broad host range of PoRV and the diversity of genotypes within circulating strains may facilitate interspecies transmission and mixed infections among different strains. During viral replication in co-infected cells, mutations and genome segment reassortment may occur, resulting in genetic variation and the emergence of novel genotype constellations, which may be further influenced by co-infection and host immune pressure [21]. Consequently, the genotypes of currently circulating strains have undergone changes [22]. Researchers across Chinese provinces are increasingly focusing their efforts on the epidemiology and evolution of PoRV, evaluating the pathogenicity of current strains. From 2018 to 2022, predominant strains in China included G5, G9, P7, P13, and P23 [6,23]. From 2021 to 2023, in conjunction with the previously predominant G9 and G5 strains, the G3 and G4 strains also exhibited widespread circulation [14]. By 2024–2025, G-types had predominantly shifted toward G9 and G4, while P13 remained the primary gene [8]. The increasing diversity of PoRV genotypes and genotype combinations has become a major trend in the current prevalence of PoRV.

PoRV genotype shifts may be associated with changes in viral pathogenicity, host adaptation, or immune escape, although such changes do not always occur. In addition, mixed infections with other common porcine diarrhea pathogens, such as PEDV, may lead to more pronounced clinical symptoms [24]. Genotypic diversity may pose a potential challenge to vaccine cross-protection. Nevertheless, the judicious selection of appropriate vaccines and the implementation of targeted control measures remain effective strategies against PoRV. Consequently, rigorous research on the genotyping, genotype constellations, and isolation of prevalent PoRV strains remains essential to understanding their epidemiological characteristics and supporting targeted prevention strategies. Furthermore, as a zoonotic pathogen, understanding and controlling PoRV epidemics also contributes to public health security in human populations [25,26]. This study investigated the epidemiological characteristics and genetic diversity of PoRV in Guangxi from 2020 to 2025. A prevalent PoRV strain, designated CH-GXGL-PoRV-3151-2021, was isolated and identified from strongly PoRV-positive clinical samples and was further subjected to whole-genome sequencing and phylogenetic analysis. By characterizing the genotypes and genotype constellations of circulating PoRV strains in this region, this study provides epidemiological data, baseline genomic information, and viral materials to support ongoing surveillance, targeted prevention strategies, and future vaccine-related research.

## 2. Materials and Methods

### 2.1. Cells, Antibodies, and Clinical Samples

The MA-104 cells used for virus isolation in this study were kindly provided by the College of Veterinary Medicine, Huazhong Agricultural University (Wuhan, China). The cells were cultured in Dulbecco’s modified Eagle medium (DMEM; Savier Bio, Wuhan, China) with penicillin–streptomycin (Savier Bio, Wuhan, China) and 10% fetal bovine serum (FBS; Biological Industries, Kibbutz Beit Haemek, Israel), under standard conditions of 5% CO_2_ and 37 °C. The following antibodies were utilized: a laboratory-prepared anti-PoRV monoclonal antibody secreted by hybridoma cells and validated for PoRV antigen detection in our laboratory as the primary antibody, fluorescein isothiocyanate (FITC)-conjugated goat anti-mouse IgG (Beyotime Bio, Shanghai, China), and 4′,6-diamidino-2-phenylindole (DAPI; Solaibao, Beijing, China).

Clinical Samples: From December 2020 to May 2025, 870 samples were collected from pigs experiencing diarrhea in Guangxi. Each sample was mixed with sterile phosphate-buffered saline (PBS; Savier Bio, Wuhan, China) in a volume ratio of 1:1. Subsequent to undergoing three freeze–thaw cycles, the samples were centrifuged at 12,000 rpm for 5 min at 4 °C. The supernatant was then collected and stored at −80 °C for further analysis.

### 2.2. Detection of PoRV and Amplification and Sequencing of VP4, VP6, and VP7 Genes

A total of 200 μL of supernatant from each collected sample was used for total RNA extraction with the Viral DNA/RNA Extraction Kit (Genstone Biotech, Beijing, China) according to the manufacturer’s instructions. PoRV nucleic acid was detected using a commercial probe-based qRT-PCR kit for porcine rotavirus detection (Biovet Biotechnology, Tianjin, China) according to the manufacturer’s instructions. After the reaction, the amplification curves were evaluated, and Ct values ≤ 35 were considered positive according to the manufacturer’s instructions. The primer and probe sequences of the commercial qRT-PCR kit were proprietary components and were not disclosed by the manufacturer. PoRV-positive samples were selected as templates for RNA extraction and amplification of target genes. The positive control was set up using a sequenced and laboratory-stored PoRV strain, while the negative control was nuclease-free H_2_O. In accordance with the manufacturer’s guidelines, the RNA was transcribed into cDNA using a HiFiScript cDNA Synthesis Kit (Cowin Biotech, Taizhou, Jiangsu, China). The 20 μL reverse transcription reaction mixture contained 4 μL of 5× RT Buffer, 4 μL of dNTP Mix, 2 μL of DTT, 2 μL of Primer Mix (PoRV-4R/6R/7R in Table 1), 1 μL of HiFiScript, and 7 μL of RNA template. The reaction was performed at 42 °C for 50 min, followed by 85 °C for 5 min.

Using specific amplification primers (Table 1), reverse transcription products (cDNA) served as templates. The amplification of target genes was performed using 2× Bench Top™ Taq Master Mix (Beiwo Medical Technology, Hangzhou, Zhejiang, China), employing VP4: PoRV-4F/4R; VP6: PoRV-6F/6R; and VP7: PoRV-7F/7R. The amplification reaction system comprised 2× Bench Top™ Taq Master Mix (15 μL), 2 μL of each primer (10 M), 5 μL of template cDNA, and nuclease-free H_2_O to 30 μL. The thermal cycling program was as follows: 95 °C for 3 min and then 35 cycles of 95 °C for 15 s, 55 °C for 20 s, 72 °C for 1 min 30 s, and 72 °C for 10 min. The amplification products were electrophoresed in 1.5% agarose gel (Savier Bio, Wuhan, China) and then imaged using a UV imaging system. Subsequently, positive PCR products that exhibited amplification bands corresponding to the expected size were selected and dispatched to Sangon Biotech (Shanghai, China) for sequencing.

### 2.3. Virus Isolation and Culture

Among the collected diarrheic clinical samples, PoRV-positive samples were further screened for virus isolation according to sample type, transport/storage conditions, and PoRV nucleic acid load. Finally, 40 strongly PoRV-positive intestinal tissue homogenates were selected for virus isolation. The clarified supernatants of strongly PoRV-positive samples were filtered through a 0.45 μm syringe filter. Trypsin (Sigma-Aldrich, St. Louis, MO, USA) was added to the filtrate at a final concentration of 20 μg/mL, and then the filtrate was incubated at 37 °C for 2 h to activate the virus. After washing with PBS twice, 500 µL of the activated filtrate was inoculated onto MA-104 cells at 37 °C in a 5% CO_2_ incubator for 2 h, thereby allowing for virus adsorption. Following the incubation period, the inoculum was removed, and the cells were washed twice with PBS. Subsequently, serum-free DMEM was added, containing a final concentration of 4 μg/mL trypsin. The culture was then maintained at 37 °C in a 5% CO_2_ incubator, with daily observation for cytopathic effect (CPE). Following the observation of 80% CPE, the cell cultures were harvested and subjected to three freeze–thaw cycles at −80 °C. Thereafter, the cultures were subjected to centrifugation at 12,000 rpm for 5 min at 4 °C. The resultant supernatant was collected and stored at −80 °C. This procedure was repeated for five consecutive passages. Cultures that exhibited stable and pronounced PoRV-specific CPE were selected and passaged continuously up to the 10th passage for preservation.

### 2.4. Virus Identification

#### 2.4.1. RT-PCR and qRT-PCR

To identify the isolated strain, viral cultures from different passages were collected, and viral RNA was extracted. PoRV nucleic acid was detected by conventional RT-PCR using the primers (PoRV-F/R) in Table 1 and by qRT-PCR using a commercial probe-based PoRV qRT-PCR detection kit (Biovet Biotechnology, Tianjin, China) according to the manufacturer’s instructions. For conventional RT-PCR, the amplified products were analyzed by agarose gel electrophoresis, and the band sizes were compared with the expected target size. For qRT-PCR, samples with typical amplification curves and Ct values ≤ 35 were considered positive.

#### 2.4.2. Immunofluorescence Assay

MA-104 cells were seeded into 12-well plates. This was followed by inoculation with the isolated PoRV strain. Following a 24 h incubation period, the culture medium was discarded. The cells were washed thrice with PBST (PBS containing 0.05% Tween-20) to reduce nonspecific binding. Subsequently, 500 μL of pre-chilled methanol was added to each well, and the plates were fixed at −20 °C for 30 min. Following fixation, the samples were washed thrice with PBS. The samples were blocked at room temperature for 1 h using blocking buffer consisting of 0.05% PBST containing 1% bovine serum albumin (BSA), washed three times with PBS, and then incubated with the PoRV monoclonal antibody at room temperature for 2 h. The cells were washed 3 times with PBS again, and then FITC-labeled goat anti-mouse IgG was added and incubated at room temperature in the dark for 1 h. After washing the object 3 times with PBS, DAPI was added and incubated at room temperature in the dark for 20 min to achieve nuclear staining. Finally, after 3 PBS washes, the stained cells were observed under a fluorescence microscope in a darkened environment.

#### 2.4.3. Tissue Culture Infectious Dose Assay (TCID_50_)

The viral titer of the isolated PoRV strain CH-GXGL-PoRV-3151-2021 was determined using the 50% tissue culture infectious dose (TCID_50_) assay. Briefly, the virus stock was serially 10-fold-diluted in maintenance medium. MA-104 cell monolayers grown in 96-well plates were inoculated with each dilution, with 10 replicate wells for each dilution. After adsorption, the inoculum was replaced with maintenance medium containing trypsin at a final concentration of 1 μg/mL, and the cells were incubated at 37 °C with 5% CO_2_. CPEs were observed daily for 7 days post-inoculation. The TCID_50_ value was calculated using the Reed–Muench method.

#### 2.4.4. Whole-Genome Sequencing

The 10th-passage viral culture of the isolated PoRV strain was collected, and total viral RNA was extracted. The extracted RNA was submitted to Suzhou Genewiz Biotechnology Co., Ltd. (Suzhou, China), for whole-genome sequencing. After assembly of the obtained sequences, sequence alignment and validation were performed using BLAST (https://blast.ncbi.nlm.nih.gov/Blast.cgi) (accessed on 15 July 2025) in NCBI.

### 2.5. Data Analysis and Visualization

Chi-square tests were performed using SPSS 26.0 to compare PoRV positivity rates among years. GraphPad Prism 9.0 was used to generate bar charts. Sequence similarity was analyzed using DNASTAR and BLAST in the NCBI database (https://blast.ncbi.nlm.nih.gov/Blast.cgi) (accessed on 15 July 2025). Phylogenetic trees were constructed in MEGA X (Version 10.2.5) using the maximum-likelihood method with the GTR + G + I model, with bootstrap values calculated from 1000 replicates, and were annotated using iTOL (https://itol.embl.de) (accessed on 10 February 2026). DeepL (https://www.deepl.com/zh) (accessed on 7 April 2026) was used only for language translation during manuscript preparation; the authors reviewed and edited the output and take full responsibility for the final content.

## 3. Results

### 3.1. Prevalence of PoRV in Clinical Samples

**Figure 1:**
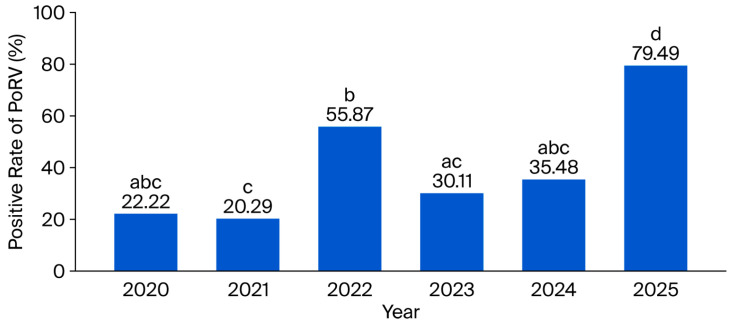
The positive rate of PoRV during 2020 to 2025. The absence of identical letters indicates a significant difference in positive rates between years (*p* < 0.05). The presence of identical letters indicates no significant difference in positive rates between years.

This study employed qRT-PCR to test 870 clinical samples collected from diarrheic pigs in Guangxi from December 2020 to May 2025. Among all clinical samples, 360 tested positive for PoRV, yielding an overall positivity rate of 41.38% (360/870). The PoRV positivity rate peaked in 2025 (79.49%, 93/121) and was the lowest in 2021 (20.29%, 14/69). Significant differences existed among years (*p* < 0.05), with an overall upward trend over time (Figure 1 and Table S1).

### 3.2. Phylogenetic Analysis of VP4, VP6 and VP7 in PoRV-Positive Samples

From the 360 PoRV-positive samples, VP4, VP6, and VP7 gene fragments were amplified and sequenced. The obtained sequences were used for two different analyses according to their length and quality. First, sequences covering the key genotyping regions were used for genotype assignment, and 86 samples with available genotype information for all three genes were included in the G/P/I genotype combination analysis. Second, only relatively complete and high-quality sequences were selected for maximum-likelihood phylogenetic reconstruction, including 82 VP4 sequences, 74 VP6 sequences, and 93 VP7 sequences.

#### 3.2.1. VP4: Clear Dominance of the P[13] Subtype, with Multiple P Subtypes Coexisting

**Figure 2:**
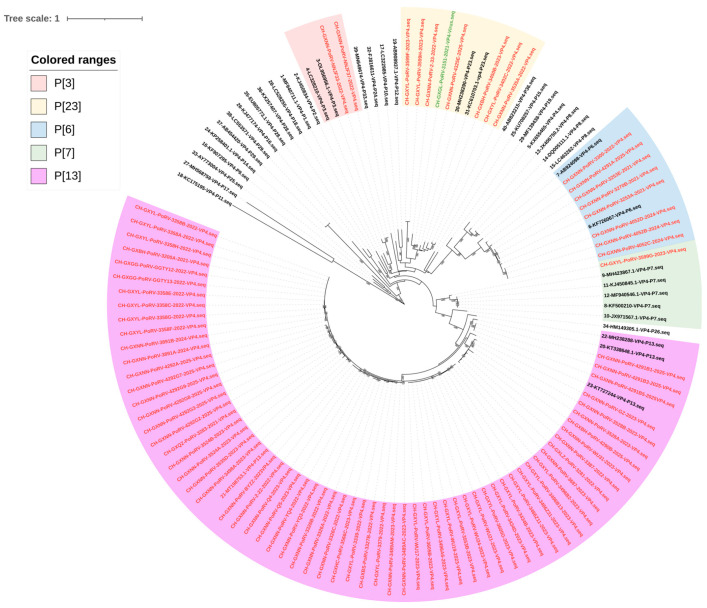
Maximum-likelihood phylogenetic tree based on VP4 gene nucleotide sequences of PoRVA strains. The tree was constructed using the GTR + G + I model. Sequences directly obtained from clinical samples are indicated in red, whereas the isolated strain CH-GXGL-PoRV-3151-2021, sequenced from passaged virus stock, is indicated in green. Bootstrap values are shown in boxes along the branches.

The maximum-likelihood phylogenetic tree analysis of the VP4 gene indicates that the obtained sequences could be classified into P[13] (76.83%, 63/82), P[7] (1.22%, 1/82), P[23] (9.76%, 8/82), P[3] (2.44%, 2/82), and P[6] (9.76%, 8/82) (Figure 2 and Table S2). All P[13] genotype strains formed an independent branch and showed the closest phylogenetic relationship with 2012–2019 Indian porcine PoRV AS/RV/SW90 (KT338648.1), Chinese swine PoRV RVA/pig/CHN/SCYB-Y1/2019/G5P13P[23] (MT198753.1), Spanish swine PoRV RVA/Pig-wt/ESP/F471/2017/G3P[13] (MH238288), and Thai pig-derived PoRV RVA/Pig-wt/THA/CMP-001-12/2012/G5P[13] (KT727244). The P[7] genotype strain forms an independent branch and is the most closely related to the 2006 Korean porcine PoRV RVA/Porcine-tc/KOR/174-1/2006/G8P[7] (MF940546.1). The P[23] genotype shares the same branch as the 2017 Italian porcine PoRV RVA/Pig-wt/ITA/7RE/2009/G9P [23] (KC610703.1) and is closely related to the 2009 Spanish porcine PoRV RVA/Pig-wt/ESP/F486/2017/G9P[23] (MH238290). The P[3] genotype samples displayed the closest relationship to the 2017 Taiwanese swine PoRV RVA/Pig-wt/TWN/106-P-001-1-0278/2017/G26P3-like (OL956956.1). Finally, the VP4 gene of the P[6]-type PoRV circulating strain forms an independent branch, most closely related to the 2013 human-origin RV RVA/Human-wt/CHN/R1954/2013/G4P[6] (KF726067).

#### 3.2.2. VP6: Primarily Genotype I5, with a Small Number of Types I1 and I14 Also Detected

**Figure 3:**
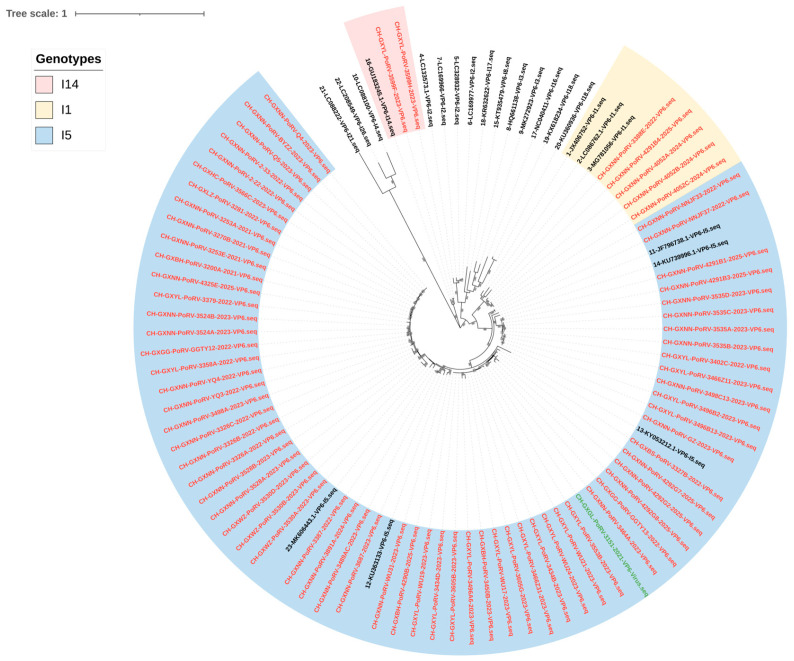
Maximum-likelihood phylogenetic tree based on VP6 gene nucleotide sequences of PoRVA strains. The tree was constructed using the GTR + G + I model. Sequences directly obtained from clinical samples are indicated in red, whereas the isolated strain CH-GXGL-PoRV-3151-2021, sequenced from passaged virus stock, is indicated in green. Bootstrap values are shown in boxes along the branches.

Based on the constructed maximum-likelihood phylogenetic tree of the VP6 gene, these sequences could be classified into I5 (90.54%, 67/74), I1 (6.76%, 5/74), and I14 (2.70%, 2/74) (Figure 3 and Table S2). The I5 genotype samples strains were closely related to the 2018 Chinese murine RV RVA/Murine-tc/CHN/SCLS-M/2018/G3P[13] (MK606443.1), the 2013 Thai human RV RVA/Human-wt/THA/CMHS-070-13/2013/G9P[19] (KU363133), the 2015 West Indies porcine PoRV RVA/Pig-wt/KNA/ET8B/2015/G5P[13] (KY053212.1), the 2006 South Korean porcine PoRV PRG9121 (JF796738.1), and the 2014 Taiwanese swine-derived PoRV RVA/Pig-wt/TWN/103-P-004-1-0575/2014/G3P19 (KU739996.1). Furthermore, the I1 genotype samples were closely related to the 1974 human-derived RV Human-tc/USA/Wa/1974/G1P[8] (JX406752), and the I14 genotype samples shared the same clade as the 2006 Canadian porcine PoRV Po/CE-M-0003/Canada/2006/G2P[27]I14 (GU183245.1).

#### 3.2.3. VP7: G5 and G9 Co-Dominant, with Multiple G Genotypes Coexisting

**Figure 4:**
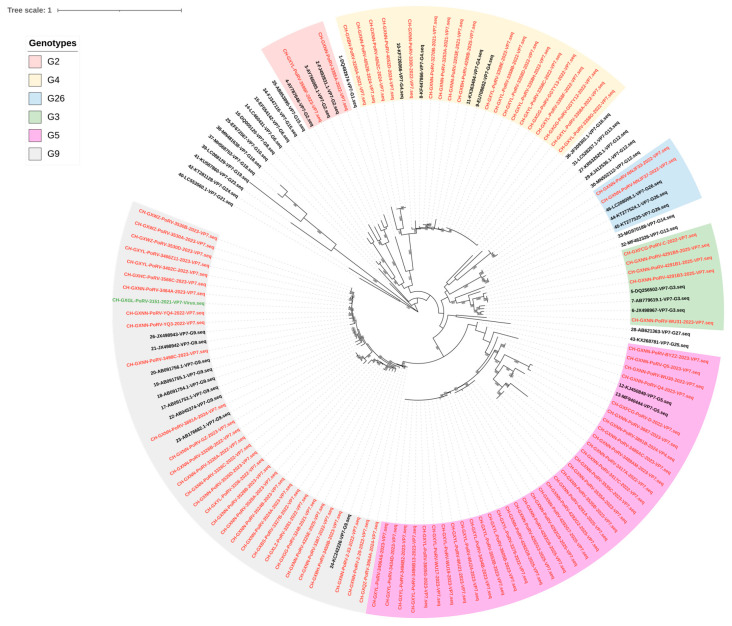
Maximum-likelihood phylogenetic tree based on VP7 gene nucleotide sequences of PoRVA strains. The tree was constructed using the GTR + G + I model. Sequences directly obtained from clinical samples are indicated in red, whereas the isolated strain CH-GXGL-PoRV-3151-2021, sequenced from passaged virus stock, is indicated in green. Bootstrap values are shown in boxes along the branches.

The maximum-likelihood phylogenetic tree of the VP7 gene showed that the obtained sequences could be classified into the following types: G5 (36.56%, 34/93), G9 (33.33%, 31/93), G4 (20.43%, 19/93), G3 (5.38%, 5/93), G2 (2.15%, 2/93), and G26 (2.15%, 2/93) (Figure 4 and Table S2). The G5 genotype sample strains share the same major subtype branch as the porcine PoRV RVA/Porcine-tc/KOR/K71/2006/G5P[7] (MF940444) from South Korea in 2006 and Spain in 2010 and RVA/Pig-tc/ESP/OSU-C5111/2010/G5P[7] (KJ450849) from South Korea in 2006 and Spain in 2010, respectively, and independently formed two distinct branches. The G9 genotype sample strains were related to the 2011 Chinese swine-derived PoRV ZJhz13-1 (JX498943), GXqz-2 (JX498942) from China in 2011, human RV LL51695 (KC242226) from China in 2006, and the Japanese JP32-4 (AB176682.1) from 2006. The G4 genotype samples exhibited high similarity to the Chinese RV GX82 (KF447866) from 2008 to 2013, RVA/Human-wt/CHN/E931/2008/G4P[6] (EU708602), RVA/Human-wt/CHN/R1954/2013/G4P[6] (EU708602), and the Vietnamese porcine PoRV RVA/Pig-wt/VNM/14226_39/VP7 (KX363404), and the 2022 strain formed an independent subclade. The G3 genotype sample strains were most closely related to the 2012 Chinese pig-derived PoRV HLJheb-1 (JX498967) and the 2006 Thai CMP096 strain. The G2 genotype sample strains displayed the closest relationship to the 2008 Chinese and 2016 Iranian porcine PoRV strains TB-Chen (AY787646) and 61/07/Ire (FJ492831.1). Finally, the G26 genotype sample strains were most closely related to the 2007 Nepal human-derived RV RVA/Human-wt/NPL/07N1760/2007/G26P[19] (LC208008.1).

#### 3.2.4. The Dominant G/P/I Genotypes Are G9P[13]I5 and G5P[13]I5

**Figure 5:**
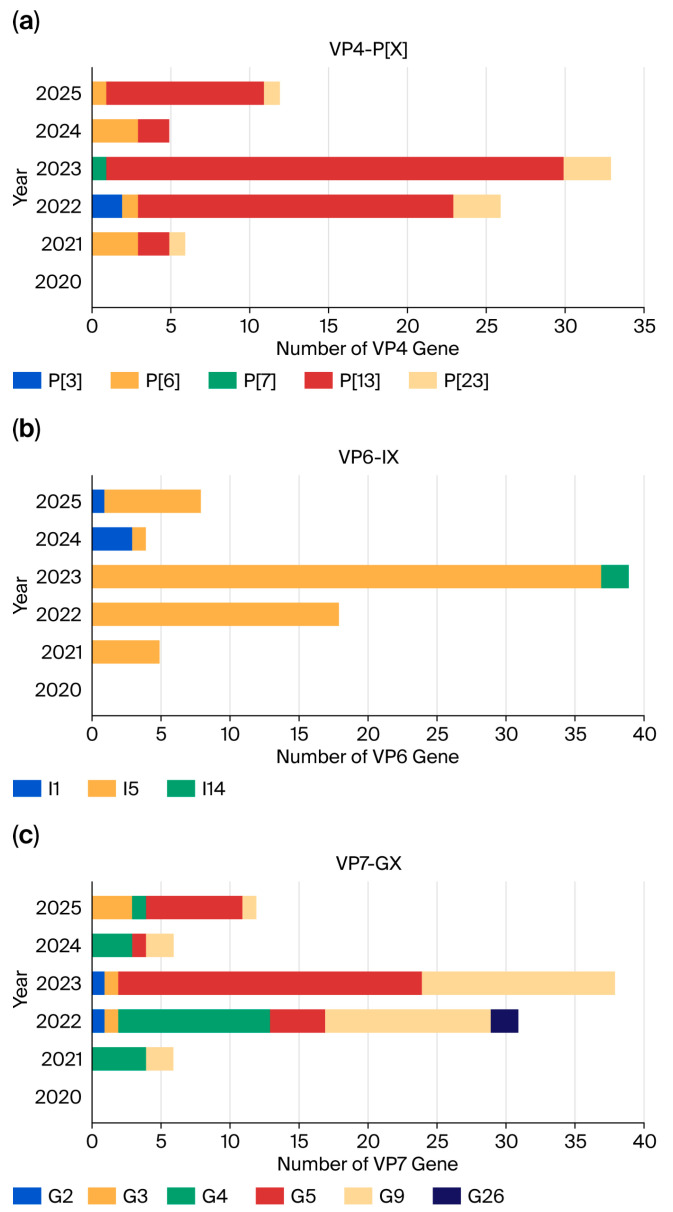
Genotypes of VP4 (**a**), VP6 (**b**), and VP7 (**c**) in circulating PoRV strains in Guangxi by year.

G/P/I genotype analysis was performed on 86 PoRV-positive samples with available genotype information for all three VP4, VP6, and VP7 genes (Figure 5 and Table S3). G9P[13]I5 (34.88%, 30/86) and G5P[13]I5 (30.23%, 26/86) were the two predominant G/P/I genotypes. Other G/P/I genotypes were detected at lower frequencies, ranging from 1.16% to 6.98%, including G9P[23]I5, G4P[13]I5, G4P[6]I5, G4P[6]I1, G5P[6]I1, G5P[23]I5, G26P[3]I1, G2P[23]I14, and G3P[13]I5. No complete G/P/I genotype information was obtained from the limited number of samples collected in 2020; therefore, the genotype distribution for 2020 is shown as 0 in the bar chart.

### 3.3. Virus Isolation and Identification

**Figure 6:**
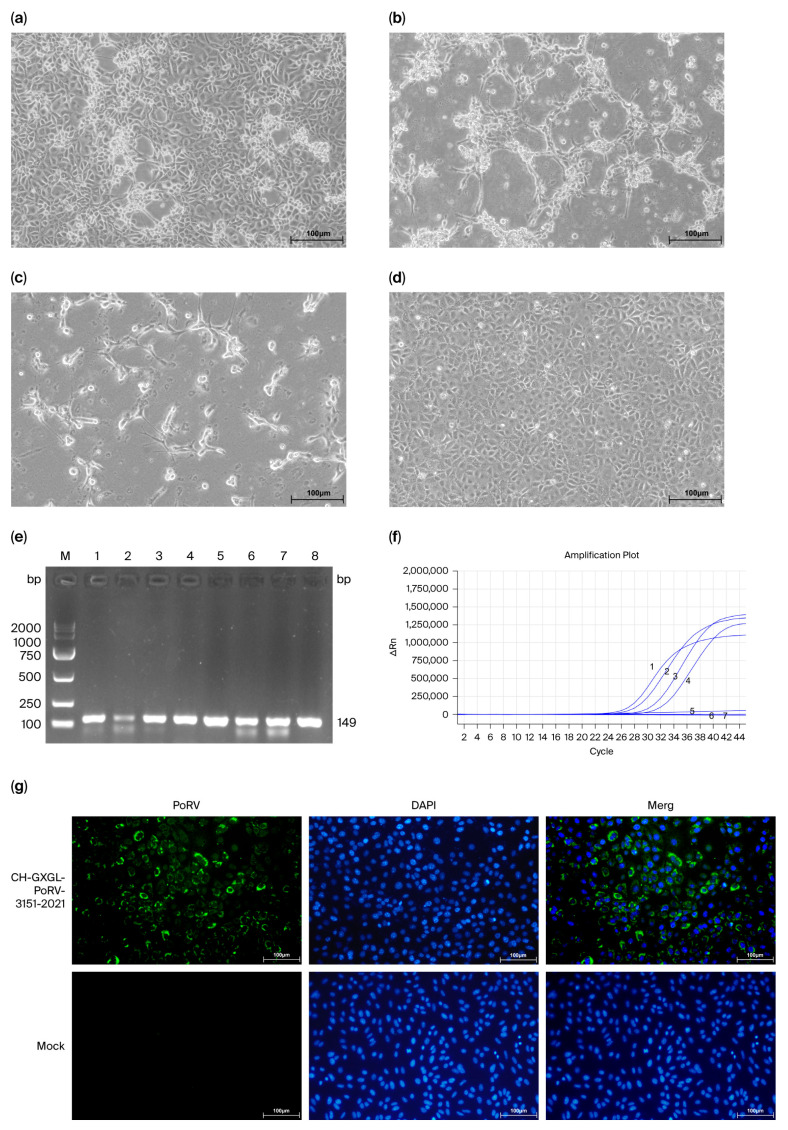
The results of virus isolation and identificaion. (**a**–**d**) Cytopathic effects (CPEs) induced by the PoRVA isolate in MA-104 cells at 16 h and 24 h post-infection under 100× magnification. Representative CPEs included cell rounding, shrinkage, detachment, and formation of network-like structures. Scale bar = 100 μm. (**e**) CH-GXGL-PoRV-3151-2021 PCR Identification Results for Different Generations in MA-104 cells. The RT-PCR detected results of positive sample and P1, P2, P3, P4, P5, P8, P10 virus cultures; (**f**) Real-time qRT-PCR detection of PoRVA RNA in the P4 (line 4, Ct = 30.51), P5 (line 3, Ct = 28.93), P8 (line 2, Ct = 26.72), P10 (line 1, Ct = 25.56) isolated virus stock and the mock-infected cell control (line 5, no Ct), DMEM-only blank cell control (line 6, no Ct) and no-template control (line 7, no Ct). According to the manufacturer’s instructions, samples with Ct values ≤ 35 were considered positive. (**g**) IFA for PoRV infection in MA-104 cells. Viral antigens were detected with a PoRV-positive serum (green), while cell nuclei were counterstained with DAPI (blue) for visualization.

Among the 870 diarrheic clinical samples, 360 were identified as PoRV-positive. After further screening based on sample type, transport and storage conditions, and PoRV nucleic acid load, 40 strongly positive intestinal tissue homogenates were selected for virus isolation. After blind passaging, one clinical specimen collected from porcine intestinal tissue in 2021 showed typical CPEs in MA-104 cells 48 h post-inoculation during the second passage (P2). The observed CPEs included marked detachment of adherent cells, cell rounding and shrinkage, and the appearance of a network-like structure. These features are indicative of PoRV infection. Subsequent to blind passage of the isolated strain from P3 to P10, the virus gradually adapted to the cells. Typical CPEs appeared in MA-104 cells 16–48 h after inoculation with the virus suspension. MA-104 cells in the mock-infected group maintained normal morphology, with no obvious signs of cytopathic effects or cell detachment (Figure 6a–d).

Nucleic acids were extracted from viral culture of positive samples from different passages, and then PoRV was detected using RT-PCR (Figure 6e) and qRT-PCR (Figure 6f). The results of the RT-PCR and agarose gel electrophoresis experiments demonstrated the presence of a prominent target band with an approximate size of 149 bp, which corresponded to the anticipated product size. Furthermore, the qRT-PCR results exhibited a characteristic S-shaped amplification curve in the target detection channel. Furthermore, a decline in Ct values was observed as the number of virus generations increased. The isolated strain was then subjected to further identification via IFA. The results demonstrated that following inoculation with the PoRV endemic strain, infected MA-104 cells exhibited specific fluorescence under fluorescence microscopy, while the mock-infected group showed no fluorescence signal (Figure 6g). These findings confirm the successful isolation of a PoRV strain that could be stably passaged from Guangxi, designated CH-GXGL-PoRV-3151-2021. The infectious titer of CH-GXGL-PoRV-3151-2021 was further determined using the TCID_50_ assay, and the P10 virus stock showed a titer of 10^7.12^ TCID_50_/mL.

### 3.4. BLAST Alignment Analysis of Genes in the CH-GXGL-PoRV-3151-2021 Strain

The 11-gene-segment sequences of CH-GXGL-PoRV-3151-2021 were subjected to BLAST analysis in NCBI database. The results indicate that the genotype of CH-GXGL-PoRV-3151-2021 is G9-P[23]-I5-R1-C1-M1-A8-N1-T1-E1-H1 (Table 2), consistent with the genetic framework of porcine rotavirus. Among these, NSP1 and NSP2 exhibited the highest degree of similarity to human RV, while other gene segments demonstrated the highest degree of similarity to PoRV. The reference strains exhibiting the highest degree of similarity to each segment were sourced from China, Taiwan, Vietnam, and Tanzania, respectively. Subsequently, the gene sequences were submitted to the NCBI GenBank database, yielding accession numbers PX874095–PX874105.

**Table 2:** Homology analysis of each gene fragment of the CH-GXGL-PoRV-3151-2021 strain.

Gene Name	Length (bp)	Type	The Closest PoRV Reference Strain in NCBI				
			Strain Name	Gene ID	Host	Country	Per. Ident (%)
VP1	1953	R1	RVA/Pig-wt/CHN/GDGYC1/2023	PP566202.1	Swine	China	97.62
VP2	1281	C1	RVA/Pig-wt/TZA/Morogoro-RP015/2019/G3P[6]	OP082211.1	Pig	Tanzania	90.55
VP3	1202	M1	RVA/Pig-wt/VNM/14226_39/VP3	KX363401.1	Sus scrofa	Viet Nam	98.44
VP4	2174	P[23]	RVA/pig/CHN/GD/10439/2023/G5P23I5	PV421676.1	Swine	China	95.66
VP6	1147	I5	CHN/GD/LG2/2022/G26I5	OQ799852.1	Porcine	China	97.99
VP7	1009	G9	GD	OR911929.1	Swine	China	97.72
Nsp1	1067	A8	RVA/Human_wt/VNM/30378/2009/G26P[19]	HG513049.1	Homo sapiens	Viet Nam	97.38
Nsp2	829	N1	Human/LL4260/China/N1	KC149928.1	Homo sapiens	China: Hebei	96.62
Nsp3	995	T1	pig-wt/CHN/CN127/2021/G12P[7]	ON989010.1	Pig	China	98.29
Nsp4	724	E1	HB2022	PV164615.1	Swine	China	98.28
Nsp5	939	H1	JSJR2023	PP100159.1	Porcine	China	92.26

### 3.5. Phylogenetic Analysis of PoRV CH-GXGL-PoRV-3151-2021

To further analyze the genetic correlations between CH-GXGL-PoRV-3151-2021 and other RVs from different regions and gene types, a nucleotide phylogenetic analysis was conducted on 11 gene segments. Because BLAST similarity and phylogenetic clustering may identify different reference strains, the closest strains described below were interpreted based on phylogenetic relationships rather than solely on the highest BLAST identity.

Phylogenetic analysis of the constructed tree revealed that the VP4 gene was typed as P[23], sharing a common branch with PoRV strains from Spain and Italy. This finding indicates close phylogenetic relationships (Figure 2). It exhibited the highest degree of homology (89.1%) with PoRV RVA/Pig-wt/ESP/F486/2017/G9P[23] (MH238290). All closely related reference strains in the phylogenetic tree were of porcine origin. The VP6 genotype is classified as type I5, consistent with the majority of PoRVs belonging to type 15. It shares the same clade as the North American PoRV RVA/Pig-wt/KNA/ET8B/2015/G5P[13] (KY053212.1) from Saint Kitts and Nevis (Figure 3), exhibiting the closest phylogenetic relationship at 95.7%. Within the same subclade, the reference strains RVA/Human-wt/THA/CMHS-070-13/2013/G9P[19] (KU363133) and RVA/Murine-tc/CHN/SCLS-M/2018/G3P[13] (MK606443.1) are from humans and murine species, respectively. The VP7 gene fragment was classified as G9, which is congruent with the 2011 Chinese PoRV and exhibits close phylogenetic affinity (Figure 4). It exhibited the highest degree of similarity (96.1%) with the 2011 PoRV GXqz-2 (JX498942). The VP7 gene clade of CH-GXGL-PoRV-3151-2021 demonstrates a high degree of phylogenetic affinity with the evolved clade of human-origin RVs within the phylogenetic tree.

**Figure 7:**
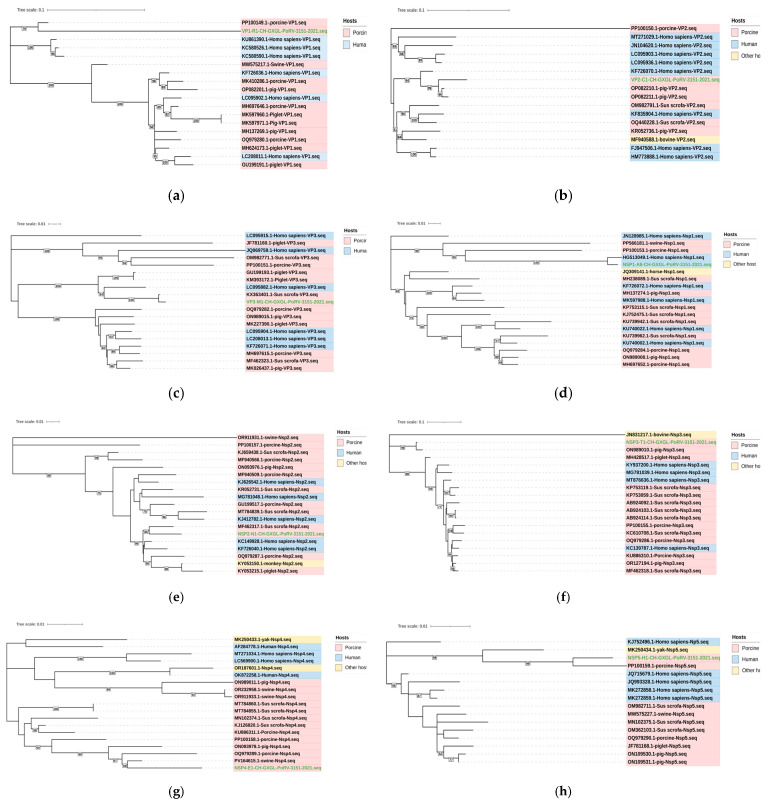
Phylogenetic analysis of the remaining eight genome segments of the isolated strain CH-GXGL-PoRV-3151-2021. The phylogenetic trees were constructed based on the VP1 (**a**), VP2 (**b**), VP3 (**c**), NSP1 (**d**), NSP2 (**e**), NSP3 (**f**), NSP4 (**g**) and NSP5 (**h**) gene sequences. The isolated strain CH-GXGL-PoRV-3151-2021 is indicated in green. The correspondingsequences of the CH-GXGL-PoRV-3151-2021 isolate are marked in green. The VP4, VP6, and VP7 segments of this isolated strain were analyzed together with the clinical sample-derived sequences in the circular phylogenetic trees shown in Figure 2, Figure 3 and Figure 4. The isolated strain is indicated in green.

The results of the phylogenetic analysis for the remaining segment genes of CH-GXGL-PoRV-3151-2021 are displayed in Figure 7. Based on phylogenetic analysis and nucleotide homology comparison, the VP1 gene (Figure 7a) belongs to the R1 genotype and forms an independent subclade. Its closest reference strain is PoRV, exhibiting an 89.0% degree of homology with the 2023 Chinese PoRV JSJR2023 (PP100149.1). The VP2 gene (Figure 7b) was identified as genotype C1 and shares the same subclade as the Tanzanian porcine strain RVA/Pig-wt/TZA/Morogoro-RP012/2019/G3P[13] (OP082210.1) with the closest phylogenetic relationship of 96.5%. Within the same VP2 subphylum, the Chinese human RV RVA/Human-wt/CHN/R1954/2013/G4P[6] (KF726070.1) also exhibits high homology at 94.4%. The VP3 gene (Figure 7c) was classified as M1 and exhibited the closest relation to PoRV RVA/Pig-wt/VNM/14226_39/VP3 (KX363401.1) in the same subclade, with 99.3% homology. A human RV RVA/Human-tc/VNM/NT0001/2007/G3P[6] (LC095882.1) has been identified in the same subclade, exhibiting a 92.8% degree of homology with the study strain.

In the non-structural genes, the NSP1 gene (Figure 7d) is classified as subtype A8, which includes the majority of PoRV strains. The reference strain within its clade is the 2009 Vietnamese human RV RVA/Human_wt/VNM/30378/2009/G26P [19] (HG513049.1), exhibiting the highest degree of homology at 98.1%. Within the NSP1 gene’s major clade, other reference strains include the 2023 Chinese PoRV RVA/Pig-wt/CHN/GDFZ/2023/G9P[23] (PP566181.1) and JSJR2023 (PP100153.1), with homologies of 84.6% and 86.9%, respectively. The NSP2 gene (Figure 7e) was genotyped as N1 and exhibited the strongest relationship with the 2016 Chinese PoRV RVA/Pig/China/LNCY/2016/G3P[13] (MF462317.1) in phylogenetic trees, with the closest phylogenetic relationship of 97.4% with the 2001 Chinese human strain Human/LL4260/China/N1 (KC149928.1). The NSP3 gene (Figure 7f) was classified as a T1 genotype and related to the 2021 Chinese PoRV pig-wt/CHN/CN127/2021/G12P[7] (ON989010.1) with 88.9% sequence identity. The NSP4 gene (Figure 7g) was identified as an E1 genotype and demonstrated the closest phylogenetic affinity to the 2019 Chinese PoRV HB2022 (PV164615.1) with the closest phylogenetic relationship (98.1%). The NSP5 gene (Figure 7h) was of the H1 type, forming an independent branch with the 2023 Chinese PoRV JSJR2023 (PP100159.1) with a homology of 94.2%.

## 4. Discussion

PoRV displays a distinctive genomic structure characterized by diverse and intricate combinations. The absence of cross-protection between different genotypes and the high mutation rate of circulating strains lead to rapid epidemic spread. Consequently, PoRV has become an increasingly important pathogen causing viral diarrhea in swine populations, resulting in substantial economic losses [1]. The objective of this study is to continue monitoring the epidemiological status and outbreak trends of PoRV in Guangxi, which will provide scientific data and experimental materials for the optimization of vaccine immunization strategies and the identification of candidate vaccine strains.

This study investigated the prevalence of PoRV in pig farms of various scales across different regions of Guangxi from December 2020 to May 2025. An evolutionary analysis of the major typing genes VP4, VP6, and VP7 was conducted on the detected circulating strains. The results of this study indicated that among a total of 870 clinical samples obtained from diarrheic pigs, 360 (41.38%) tested positive for PoRV, indicating an overall positivity rate. This result was higher than the positivity rates of 37.1% during 2021–2023 in Guangxi [23] and 25.9% during 2022–2023 across five Chinese provinces [27]. The overall PoRV positivity rate showed an overall upward trend from 2020 to 2025, aligning with the findings reported by Zhang F. et al. [14] and He S. [23]. This finding suggests a high prevalence of PoRV infection in diarrheic pigs in Guangxi over recent years. PoRV remains the predominant viral pathogen responsible for piglet diarrhea in Guangxi’s swine industry. To this end, there is an imperative for the ongoing enhancement of PoRV detection and surveillance across all levels of pig farms, which is crucial to providing a scientific foundation that enables the precise targeting of prevention and control measures against PoRV infection.

In PoRV, the VP4 gene has been identified as a crucial mediator of viral infection, while the VP7 gene has been confirmed as an ideal target for RV vaccine development. The VP6 gene, in particular, constitutes the primary component of the capsid surface particles in viral particles. These three genes, respectively, determine the P-type, G-type, and serotype grouping of PoRV, all of which are key antigens of PoRV. Given the propensity of PoRV to exhibit diverse genotype combinations among its segments, heterologous immunity is imperative for combating RV infection [27]. In this study, 82 VP4 sequences, 74 VP6 sequences, and 93 VP7 sequences met the quality criteria for phylogenetic analysis. In addition, 86 samples with available genotype information for VP4, VP6, and VP7 were included in the G/P/I genotype analysis. Not all positive samples were successfully amplified for all three genes, likely due to the non-universal applicability of amplification primers across different variants of the same gene and varying viral loads in samples. The present study identified five P types (P[3], P[6], P[7], P[13], and P[23]), three I types (I1, I5, and I14), and six G types (G2, G3, G4, G5, G9, and G26), which encompass common genotypes identified in recent epidemiological investigations in PoRV (P[6], P[13], P[23], G3, G4, G5, and G9) alongside less prevalent subtypes (P[3], P[7], G2, and G26) [14,21,22,23,24,25,27,28,29]. VP6 demonstrates relative conservation, predominantly belonging to the I5 type, with approximately 10% being I1 type. Some strains exhibit high homology with human RV (primarily subtyped as I1 and I2 [30]), while the I14 type is mainly found in PoRV group C (RVC) [31]. In this study, the presence of the I14 VP6 genotype in two positive samples suggests the circulation of genetically diverse VP6 genotypes. Among the strains successfully amplified for the VP4, VP6, and VP7 genes, 11 distinct G/P/I genotype combinations were identified, including G9-P[13]-I5, G9-P[23]-I5, G5-P[13]-I5, G5-P[6]-I1, G5-P[23]-I5, G4-P[13]-I5, G4-P[6]-I5, G4-P[6]-I1, G26-P[3]-I1, G2-P[23]-I14, and G3-P[13]-I5. The predominant genotypes exhibited variability across the study period. VP4 predominantly exhibited the P[13] type, VP6 predominantly showed the I5 type, and VP7 was dominated by the G9 and G5 types. The primary combinations that were identified were G9-P[13]-I5 and G5-P[13]-I5, as well as G4-P[6]-I5, and the G9-P[23]-I5 genotype demonstrated consistent stability. These findings are consistent with recent genetic evolution analyses of PoRV across different regions in China [22]. Homology analysis revealed that some genes showed high similarity to human RV (I1) and C-type PoRV (I14), suggesting potential risks of cross-species transmission or reassortment [32]. The emergence of complex genotypes may result in diminished cross-protective immunity from vaccines, a phenomenon that could underlie the present limited vaccine efficacy.

Despite the relatively high detection rates of PoRV in fecal samples from diarrheic pigs, its unique particle structure imposes limitations on in vitro culture, which is usually performed using susceptible intestinal epithelial or kidney cells. Furthermore, the high trypsin dependency of G9-type PoRV results in low isolation efficiency when fecal matter or intestinal tissue homogenates from diarrheic pigs are directly inoculated into cells. Research has demonstrated that PoRV demonstrates higher levels of infectivity in MA-104 cells compared with IPEC-J2 and HT-29 cells. Furthermore, trypsin pretreatment of samples has been shown to enhance PoRV isolation efficiency [33]. However, elevated trypsin concentrations have been observed to result in a decline in cell viability and the manifestation of morphological alterations, which makes it difficult to distinguish between the morphological changes induced by trypsin and the CPE caused by PoRV formation. Therefore, prior to isolating the virus, the maximum tolerable concentration of trypsin was determined for the MA-104 cell line used in the experiment. In this study, only one circulating PoRV strain from Guangxi was isolated from 40 clinical samples with high viral loads; it was designated CH-GXGL-PoRV-3151-2021. During the virus isolation process, the trypsin activation time of the virus suspension was appropriately extended. After exploring different isolation systems, it was found that treating the medium with 20 μg/mL trypsin for 2 h to activate the virus, followed by 1.5–2 h of virus incubation, appeared to be the most effective condition in this study. Additionally, a concentration of 4 μg/mL trypsin was used in the experimental cell culture, and sham-infected wells were included in the isolation process. Under these conditions, which allowed for the differentiation of trypsin-induced cellular morphological changes, PoRV was successfully isolated from clinical samples. These findings indicate that the correct selection of susceptible cells and ensuring their growth status to ensure tolerance to the concentration of trypsin used during viral culture, along with adequate pretreatment and activation of the viral solution from infected cells, are crucial to the successful isolation and culture of rotavirus. This study provides insights into the isolation of PoRV strains.

The genetic evolutionary analysis of PoRV-related gene sequences is regarded as the gold standard for the study of viral evolution. In order to comprehend the genetic composition of the Guangxi PoRV strain CH-GXGL-PoRV-3151-2021, which is isolated to the region, whole-genome second-generation sequencing was performed on the strain. This analysis covered a total of 11 gene segments. Each of the 11 gene segments was subjected to BLAST alignment analysis against the NCBI database, followed by phylogenetic analysis using software such as MEGA X (Version 10.2.5) and MegAlign (Version 7.1.0). According to the emerging RV genotyping system, the complete genotype of this strain is G9-P[23]-I5-R1-C1-M1-A8-N1-T1-E1-H1. These typing results were consistent with the PoRV genotypes that had been isolated by other researchers in recent years [33,34,35]. It has been hypothesized that this particular strain type may be more amenable to isolation than other PoRV types, potentially exhibiting higher levels of infectivity or replication capacity. G9P[23] strains have been demonstrated to exacerbate PEDV infection in co-infected pigs, exhibiting a more pronounced promoting effect than G5P[7] strains [24]. Genetic analysis of CH-GXGL-PoRV-3151-2021 indicated that its NSP1 and NSP2 gene segments shared the highest nucleotide similarity with human rotaviruses, suggesting a possible porcine–human reassortant. However, the reassortment pattern, parental origins and transmission direction require further verification using recombination detection tools and virological assays. The most similar reference strains to CH-GXGL-PoRV-3151-2021 originate from different regions and countries. These results indicate that during its circulation and evolution, PoRV exhibits extensive geographical spread and transmission, with strains readily interacting and undergoing reassortment. In the phylogenetic tree, VP2 and VP3 demonstrated comparable affinity with human RV, while NSP3 exhibited a similar association with bat RV. Although CH-GXGL-PoRV-3151-2021 is not a predominant genotype, its complete genome sequence underscores the genetic diversity of PoRV in the region, including potential porcine–human reassortment events, providing a tangible case for studying viral evolution and offering research material for studying the pathogenicity of prevalent PoRV strains in Guangxi.

This study has several limitations. First, the clinical samples were mainly submitted diagnostic samples collected from different pig farms during routine diagnostic testing and surveillance. Therefore, some background information, including pig age, production stage, breeding purpose, sample quality, and storage history, was incomplete or unavailable. In addition, sample collection was concentrated in specific regions and time periods. Although preliminary comparisons of PoRV positivity rates across different cities in Guangxi and different seasons were performed, these results are not presented in the text because the uneven distribution of samples could not reliably support further association analyses with circulating genotypes. Thus, the positivity rate data should be interpreted with caution and are suitable only for preliminary observations of annual PoRV infection intensity. Moreover, other common enteric pathogens associated with porcine diarrhea were not comprehensively analyzed, and potential co-infections could not be excluded.

Second, this study focused on genotyping and preliminary phylogenetic analysis of PoRV strains circulating in Guangxi in recent years. However, detailed analyses of nucleotide or amino acid mutations associated with pathogenicity, antigenicity, and biological function were not performed, and such analyses should be addressed in future targeted studies. Finally, only one PoRV strain was successfully isolated. Although the whole-genome sequencing of CH-GXGL-PoRV-3151-2021 was completed, data from a single strain are insufficient to support in-depth conclusions regarding its evolutionary origin, reassortment history, epidemiological drivers, reassortment frequency, evolutionary trends, or biological function. Therefore, the prevalence and epidemiological significance of this genomic constellation in pig populations remain to be further clarified. In addition, Figure 6 is based on representative observations at selected time points and is intended to support virus isolation and identification rather than to fully characterize the replication kinetics of the isolated strain. Nevertheless, PoRV strains with the same genomic constellation have been reported in different regions of China in recent years, suggesting that continued surveillance of this genotype is warranted. Therefore, the complete genome information obtained in this study provides useful baseline data for future multi-center and long-term surveillance studies, as well as for further investigations of pathogenicity, antigenic variation, biological function, and disease prevention.

## 5. Conclusions

The PoRV positivity rate among the tested diarrheic samples in Guangxi from 2020 to 2025 showed an overall increasing trend during the study period. The genotypes exhibited a high degree of complexity and diversity, with the predominant G, I, and P genotypes being G5/G9, I5, and P[13], respectively. The most common genotype combinations were G9-P[13]-I5, G5-P[13]-I5, and G4-P[6]-I5. The potential for cross-species transmission underscores the necessity for caution and vigilance in this context. A locally circulating PoRV strain, designated as CH-GXGL-PoRV-3151-2021, was successfully isolated from PoRV-positive clinical samples in Guangxi. The complete genome of this strain exhibits the genotype constellation G9-P[23]-I5-R1-C1-M1-A8-N1-T1-E1-H1. BLAST alignment and phylogenetic analysis revealed a high degree of similarity between this strain’s genome and the genomes of human RV and PoRV. This finding suggests the possibility of genetic reassortment or exchange during the evolutionary process, along with the potential for interspecies transmission risk. This study provides epidemiological data and an isolated strain resource for subsequent studies on the genetic characteristics, antigenic variation, and biological properties of circulating PoRV strains in Guangxi, which may provide reference material for future vaccine-related research.

## Figures and Tables

**Table 1:** Specific amplification primers.

Primer	Sequence	Length (bp)
PoRV-4F	5′-GGCTTCGCTCATTTATAGAC-3′	802
PoRV-4R	5′-GTTGCATTGCATTTCTTTCC-3′	
PoRV-6F	5′-GGCTTTTAAACGAAGTCTTC-3′	1356
PoRV-6R	5′-GGTCACATCCTCTCACTA-3′	
PoRV-7F	5′-GGCTTTAAAAGAGAGAATTTCCGTCTGG-3′	1062
PoRV-7R	5′-GGTCACATCATACAATTCTAATCTAAG-3′	
PoRV-F	5′-AAGCACGATTTGGAACCATCG-3′	149
PoRV-R	5′-AGTAAGTCCAACTGTGGCATGA-3′	

## Data Availability

The raw data supporting the conclusions of this article will be made available by the authors on request.

## Supplementary Materials

The following supporting information can be downloaded at: https://www.mdpi.com/article/10.3390/vetsci13070631/s1, Table S1. The positivity rate of PoRV during 2020 to 2025. Table S2. Reference sequences for Figure 2, Figure 3, Figure 4 and Figure 7. Table S3. Genotypes of VP4, VP6, VP7 and G/P/I genotype combinations in circulating PoRV strains in Guangxi.
